# Case Report: A case of ultra-late recurrence of *KIF13A-RET* fusion non-small cell lung cancer response to selpercatinib

**DOI:** 10.3389/fonc.2023.1178762

**Published:** 2023-04-25

**Authors:** Ha-Young Park, Joo-Heon Park, Myung-Geun Shin, Seung Jung Han, Yong-Sok Ji, Hyung-Joo Oh, Young-Chul Kim, Taebum Lee, Yoo-Duk Choi, In-Jae Oh

**Affiliations:** ^1^ Department of Internal Medicine, Chonnam National University Medical School and Hwasun Hospital, Hwasun, Republic of Korea; ^2^ Departments of Laboratory Medicine, Chonnam National University Medical School and Hwasun Hospital, Hwasun, Republic of Korea; ^3^ Dxome Co. Ltd., Seongnam-si, Gyeonggi-do, Republic of Korea; ^4^ Department of Ophthalmology, Chonnam National University Medical School and Hospital, Gwangju, Republic of Korea; ^5^ Department of Pathology, Chonnam National University Medical School and Hospital, Gwangju, Republic of Korea

**Keywords:** non-small cell lung cancer, selpercatinib, *RET* fusion, choroidal metastasis, recurrence

## Abstract

**Background:**

Cancer recurrence remains a significant problem, and most postoperative recurrences of non-small cell lung cancer (NSCLC) develop within 5 years after resection. We present a rare case of ultra-late recurrence of NSCLC accompanying choroidal metastasis with *KIF13A-RET* fusion 14 years after the definitive surgery.

**Case description:**

A 48-year-old female patient who had never-smoked presented with decreased visual acuity. She had been treated with right upper lobe lobectomy followed by adjuvant chemotherapy 14 years prior. Fundus photographs revealed bilateral choroidal metastatic lesions. Positron emission tomography-computed tomography (PET-CT) scans showed extensive bone metastases and focal hypermetabolism in the left uterine cervix. An excision biopsy of the uterus showed primary lung adenocarcinoma with immunohistochemistry of TTF-1+. Plasma next-generation sequencing (NGS) identified the presence of *KIF13A-RET* fusion. After 6 months of selpercatinib therapy, PET-CT revealed a partial response for bone and uterine metastasis and stable disease for choroidal lesions.

**Conclusion:**

In this case report, we are reporting a rare case of ultra-late recurrence of NSCLC in a patient with choroidal metastasis. Furthermore, the diagnosis of NSCLC with *RET* fusion was based on liquid-based NGS rather than tissue-based biopsy. The patient showed a good response to selpercatinib, which supports the efficacy of selpercatinib as a treatment for *RET*-fusion-positive NSCLC with choroidal metastasis.

## 1 Introduction

Lung cancer is the most common cause of cancer-related deaths worldwide. Complete surgical resection including adjuvant therapy is the most effective treatment for non-small cell lung cancer (NSCLC). Recurrence remains a major problem, and most postoperative recurrences of NSCLC develop within 5 years after resection. However, ultra-late recurrence developing more than 10 years after resection is extremely rare (1).

As many chemotherapeutic and targeted agents were developed, the survival of advanced NSCLC was extended. However, this improvement was not indicated for rare genetic alterations such as *RET* fusion, which is detected in 1% to 2% of NSCLC cases (2). Selpercatinib is a novel, adenosine triphosphate competitive, highly selective small-molecule inhibitor of *RET* kinase. As this medicine was approved for metastatic NSCLC with *RET* alteration, treatment response and survival have improved (3).

In this report, we present a rare case of ultra-late recurrence of NSCLC accompanying choroidal metastasis with *KIF13A-RET* fusion 14 years after the definitive surgery.

## 2 Case presentation

**Figure 1:**
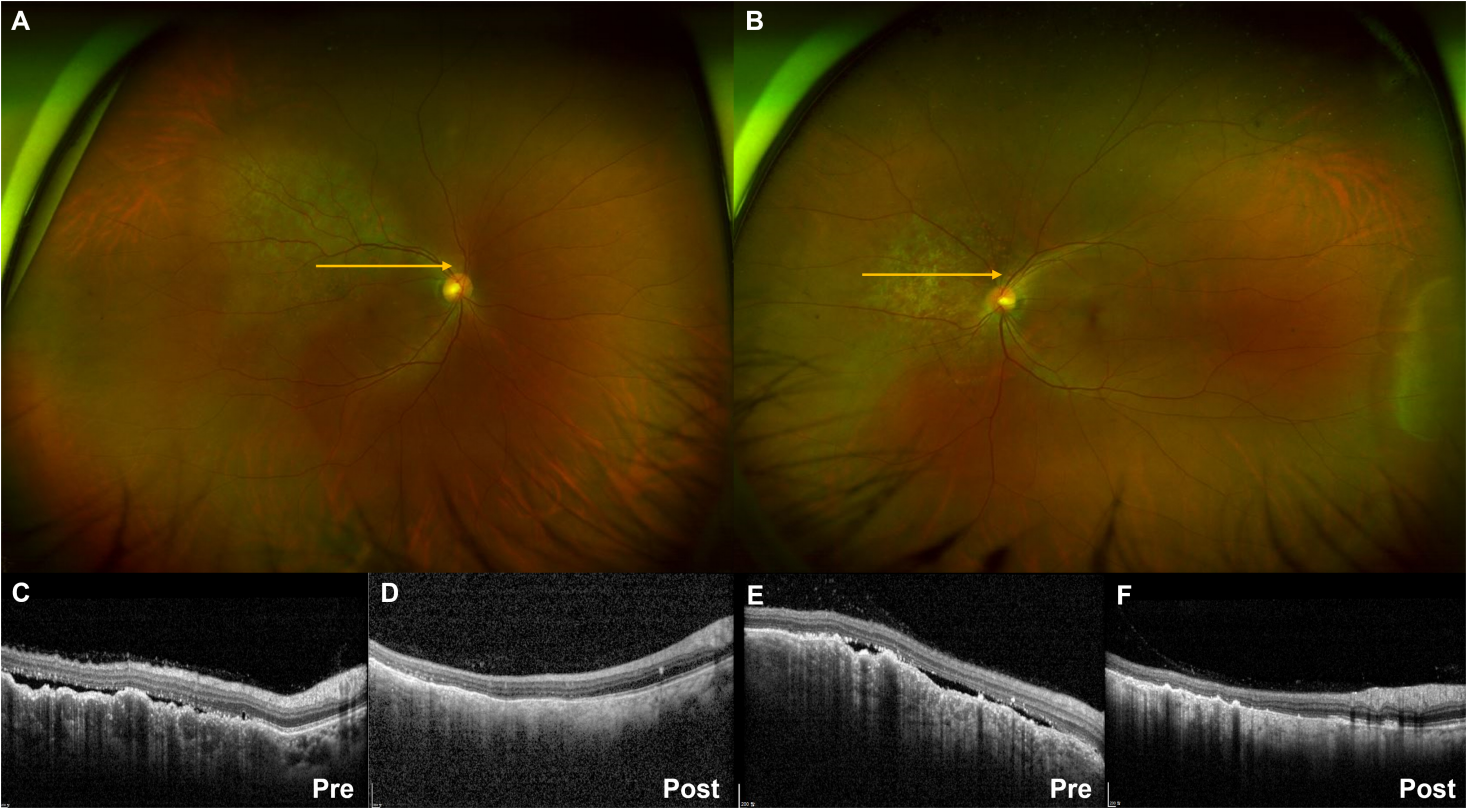
Ultra-widefield fundus photography showing pigmented choroidal elevated lesions in the right **(A)** and left **(B)** eye. Yellow arrows indicate the optical coherence tomography (OCT) scan direction. The subretinal fluid and choroidal folds on OCT images before treatment **(C, E)** showed improvement after selpercatinib treatment **(D, F)**.

**Figure 2:**
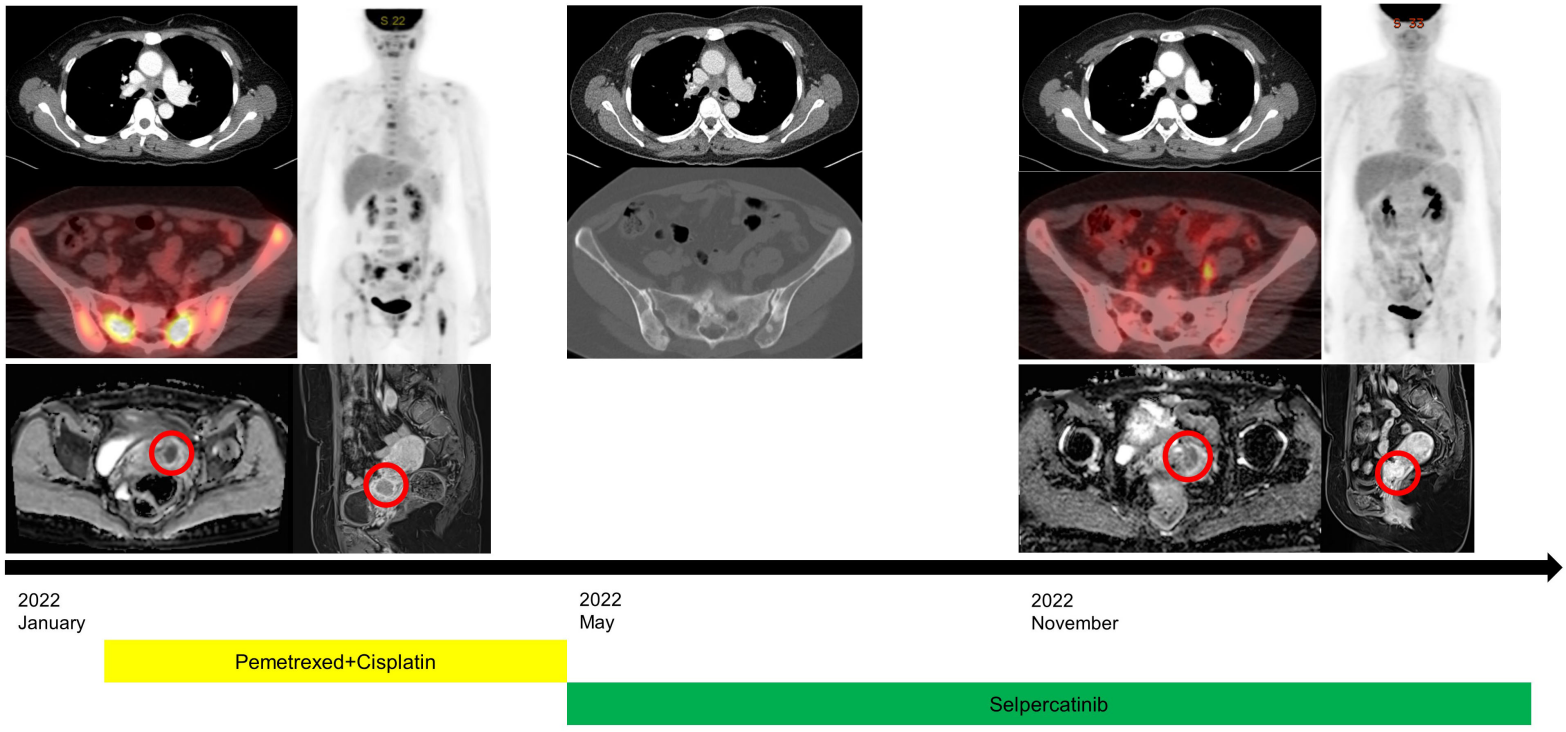
Clinical course with radiological images at initial diagnosis, the beginning of selpercatinib treatment, and after treatment. Positron emission tomography-computed tomography revealed a partial response for multiple bone metastases and slightly decreased size of uterine cervical metastasis (red circles) on pelvic magnetic resonance image.

**Figure 3:**
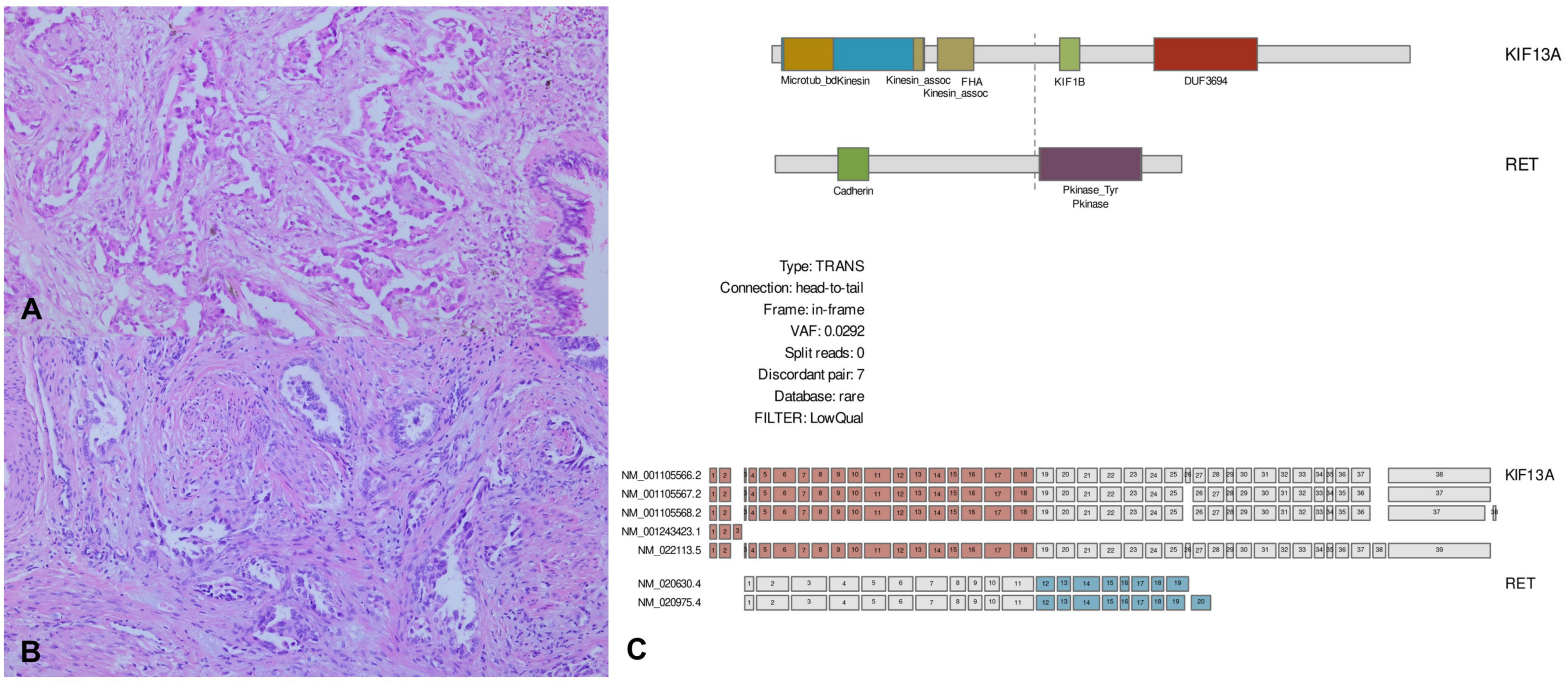
Pathological images and next-generation sequencing (NGS). **(A, B)** Microscopic findings of the lung surgical specimen and uterine biopsy. Lung adenocarcinoma exhibits an acinar growth pattern in invasive component, which is similar to the histology seen in uterine biopsy (H&E, 100×). **(C)** Plasma NGS shows *KIF13A-RET* rearrangement with a variant allele fraction of 0.0292%. Library preparation and target capture were performed using a commercial kit and custom target gene panel (Dxome). Massive parallel sequencing was performed using a high-throughput sequencing machine (Illumina).

A 48-year-old female patient who had never smoked presented with decreased visual acuity. On April 2008, she received right upper lobe lobectomy to treat lung adenocarcinoma with clinical stage IA (T1N0M0), according to the *TNM Classification of Malignant Tumors*, 7th edition. Her pathological stage was Stage IIIB (T4N1M0), and she received four cycles of adjuvant chemotherapy with paclitaxel and cisplatin. On August 2015, there was no evidence of disease, and regular cancer surveillance was terminated. In December 2021, she revisited our hospital with decreased visual acuity for about 3 months. Fundus photographs revealed bilateral choroidal metastatic lesions (**Figures 1A–C, E**), but computed tomography showed no definitive lung mass. Positron emission tomography-computed tomography (PET-CT) scans showed extensive bone metastatic lesions and focal hypermetabolism in the left uterine cervix (**Figure 2**). Magnetic resonance image (MRI) revealed two tiny metastases in the left middle frontal gyrus and lingual gyrus on brain. On pelvic MRI, there was a 6.0 × 4.3 × 2.1 cm heterogeneously enhancing mass in the endocervical canal. On January 2022, pathology from the excision biopsy of the uterus showed primary lung adenocarcinoma with an immunohistochemistry of TTF-1+, CK7+ and Ki-67+ without PD-L1 expression, which were similar findings as those from previously resected tissue in 2008 (**Figures 3A, B**). The real-time polymerase chain reactions to epidermal growth factor receptor and *ROS* proto-oncogene 1 receptor tyrosine kinase (*ROS1*) were negative, and anaplastic lymphoma kinase (*ALK*) fluorescence *in situ* hybridization was also negative. Subsequently, she received four cycles of chemotherapy with pemetrexed and cisplatin, and stable disease was observed. However, she suffered anaphylaxis during the 2^nd^ cycle of cisplatin injection, so it was discontinued. We performed next-generation sequencing (NGS) of the uterine tissue and plasma, and then identified the presence of the *KIF13A-RET* fusion gene from the plasma sample (**Figure 3C**). Tissue NGS showed *MDM2* copy gain (tier II) without *RET* fusion. The patient began treatment with selpercatinib 160 mg twice daily, based on her plasma result thanks to the Named Patient Program. She also received intravitreal injection of bevacizumab. After 6 months of *RET*-targeted therapy, PET-CT revealed a partial response for multiple bone metastases (**Figure 2**). Optical coherence tomography showed improvement of the subretinal fluid and choroidal folds after treatment (**Figures 1D, F**). She is continuing selpercatinib medication for more than 9 months without any safety issues.

## 3 Discussion

This case exhibits several rare clinical features. First, choroidal metastasis is a relatively rare complication in lung cancer, but lung cancer is the second most common tumor that causes metastasis development in the choroid (4). In choroidal metastasis, adenocarcinoma is the most common histology, and left eye involvement is more common than right eye involvement. Patients who received systemic chemotherapy for choroidal metastasis tend to be younger than median age (4). Choroidal metastasis typically presents only when another organ system is affected by metastasis, and is usually diagnosed before or early after diagnosis of primary lung cancer (5).

Second, our case developed recurrence 14 years after surgical resection. Few cancers tend to recur more than 10 years after resection, which is called ‘ultra-late recurrence.’ There are only a few reported cases of ultra-late recurrence, and those is not well known. Dai et al. studied cases of ultra-late recurrence that developed in 2.5% of total patients who had surgical resection for NSCLC. Their histologic type was adenocarcinoma, and lymphatic invasion significantly influenced ultra-late recurrence (1). Matsuo et al. reported a very rare case of recurrent *EML4-ALK* fusion-positive lung adenocarcinoma with an interval of 16 years after curative surgery (6). Therefore, patients with *ALK* and *RET* gene rearrangement may need long-term follow-up of more than 15 years after surgery.

Third, NGS was performed on both tissue and plasma specimens, and *KIF13A-RET* fusion was found only in plasma in our case. Liquid biopsy using NGS is a rapidly evolving application because it is convenient and less invasive. Compared to tissue biopsies, liquid biopsies can relieve patient’s discomfort and overcome tumor heterogeneity (7). In our case, thirty nanograms of DNA were used for the experiments, and library preparation was performed using the DxSeq ctDNA Pan100 for the Illumina Platform Kit (Dxome, Seoul, Republic of Korea) according to the manufacturer’s instructions. Paired-end sequencing was performed on the NovaSeq 6000 System (Illumina) with a 300-cycle protocol targeting at least 150 million sequencing reads and >20,000× mean depth.

*RET* is a transmembrane glycoprotein receptor-tyrosine kinase that is encoded by the *RET* proto-oncogene on chromosome 10. This kinase helps kidneys and the enteric nervous system during embryogenesis (8). *RET* fusion is an oncogenic driver that occurs in 1% to 2% of NSCLC patients and has a high occurrence rate in young women, non-smokers, and lung adenocarcinoma (9). *KIF5B-RET* (68%) is the most frequently observed *RET* fusion in NSCLC, followed by *CCDC6-RET* (12%), and *KIF13A-RET* fusion was classified as one of the rare noncanonical fusion (10). There were two available kinase inhibitors for *RET* fusions; selpercatinib and pralsetinib. Selpercatinib is the first-developed *RET* inhibitor. In a phase 1/2 study (LIBRETTO-001, ClinicalTrials.gov Identifier: NCT03157128), the objective response rate (ORR) was 64%, and median progression-free survival was 16.5 months in patients who had previously received platinum-based chemotherapy (3). Selpercatinib treatment showed relatively high intracranial activity: ORR was 82% and the intracranial disease control rate was 100%, and at 6 months, 79% of patients were intracranial progression/death-free (11). Besides, a phase III study (LIBRETTO-432, ClinicalTrials.gov Identifier: NCT04819100) about selpercatinib as an adjuvant therapy after surgery or radiation is enrolling study subject (12). Pralsetinib showed overall responses was 61% of patients who had previous platinum-based chemotherapy in a phase 1/2 study (13).

## 4 Conclusion

In this report, we treated a rare case of ultra-late recurrence of NSCLC in a patient with choroidal metastasis. Furthermore, the patient was diagnosed with NSCLC with *RET*-fusion according to liquid-based NGS, rather than tissue-based biopsy. The patient showed a good response to selpercatinib, which supports the efficacy of selpercatinib for treating *RET* fusion-positive NSCLC with choroidal metastasis.

## Data availability statement

The datasets presented in this article are not readily available because of ethical/privacy restrictions. Requests to access the datasets should be directed to the corresponding author.

## Ethics statement

The studies involving human participants were reviewed and approved by the institutional review board of the Chonnam National University Hwasun Hospital. Written informed consent was obtained from the patient for the publication of this case report, including any potentially-identifying images or data.

## Author contributions

Conceptualization: H-YP and I-JO. Data curation: H-YP, J-HP, SH, TL and I-JO. Analysis and interpretation of data: H-YP, J-HP, TL and I-JO. Funding acquisition: I-JO. Writing-original draft: H-YP, Y-SJ and, TL. Writing-review & editing: Y-DC and I-JO. Manuscript approval: all authors. All authors contributed to the article and approved the submitted version.
